# Study of the Thermal Phase Transition of Poly(*N,N*-diethylacrylamide-*co*-*N*-ethylacrylamide) Random Copolymers in Aqueous Solution

**DOI:** 10.3390/polym16111575

**Published:** 2024-06-02

**Authors:** José Javier Coca-Hidalgo, Maricarmen Recillas-Mota, Daniel Fernández-Quiroz, Jaime Lizardi-Mendoza, Carlos Peniche-Covas, Francisco M. Goycoolea, Waldo M. Argüelles-Monal

**Affiliations:** 1 Centro de Investigación en Alimentación y Desarrollo, Hermosillo 83304, Mexico; jcoca221@estudiantes.ciad.mx (J.J.C.-H.); mrecillas@ciad.mx (M.R.-M.); jalim@ciad.mx (J.L.-M.); 2 Departamento de Ingeniería Química y Metalurgia, Universidad de Sonora, Hermosillo 83000, Mexico; daniel.fernandez@unison.mx; 3 Facultad de Química, Universidad de La Habana, La Habana 10400, Cuba; cpeniche2015@yahoo.com; 4 School of Food Science and Nutrition, University of Leeds, LS2 9JT Leeds, UK; fmartin.goycoolea@um.es

**Keywords:** poly(*N,N*-diethylacrylamide), poly(*N*-ethylacrylamide), coil-to-globule phase transition, hydrodynamic radius, ζ-potential, thermosensitive copolymers

## Abstract

*N*-alkyl-substituted polyacrylamides exhibit a thermal coil-to-globule transition in aqueous solution driven by an increase in hydrophobic interactions with rising temperature. With the aim of understanding the role of *N*-alkyl substituents in the thermal transition, this study focuses on the molecular interactions underlying the phase transition of poly(*N,N*-diethylacrylamide-*co*-*N*-ethylacrylamide) random copolymers. Poly(*N,N*-diethylacrylamide) (PDEAm), poly(*N*-ethylacrylamide) (PNEAm), and their random copolymers were synthesized by free radical polymerization and their chemical structure characterized spectroscopically. It was found that the values of the cloud-point temperature increased with PNEAm content, and particle aggregation processes took place, increasing the negative charge density on their surface. The cloud-point temperature of each copolymer decreased with respect to the theoretical values calculated assuming an absence of interactions. It is attributed to the formation of intra- and interchain hydrogen bonding in aqueous solutions. These interactions favor the formation of more hydrophobic macromolecular segments, thereby promoting the cooperative nature of the transition. These results definitively reveal the dominant mechanism occurring during the phase transition in the aqueous solutions of these copolymers.

## 1. Introduction

*Smart* polymers are materials that exhibit large variations in their properties in response to small changes in the environment. These materials can be subjected to reversible changes in their physicochemical properties in response to a single stimulus (or multiple stimuli), such as a variation in temperature [1,2,3], pH [4,5], electric or magnetic fields [6], light intensity [7,8], and biomolecules [9], among others. Some of the responses observed in *smart* polymers are swelling, collapse, or sol–gel transitions depending on the physical state of the chains [10]. These polymers have been used in the development of advanced materials with multiple applications, especially in the biomedical field [11]. Among them are applications in drug delivery systems, tissue engineering, biosensors, bioimaging, injectable hydrogels—such as intraocular or intramuscular—nanocarriers, self-healing hydrogels, etc. [11,12,13,14,15,16,17,18,19].

Thermosensitive polymers are among the most widely studied smart materials. In fact, since the discovery of the volume phase transition in the late 1970s by the pioneering works and theoretical framework developed by Prof. Tanaka [20,21,22], a great deal of knowledge has been gained about these materials and the nature of the phase transition. He observed the discontinuous changes that take place under some specific conditions by polyacrylamide gels, particularly poly(*N*-isopropylacrylamide). It is currently accepted that the phase transition in most thermosensitive hydrogels involves nonionic interactions such as van der Waals, hydrophobic, and hydrogen bonding, jointly with ionic coulombic interactions if polyelectrolytes are included.

**Figure 1:**
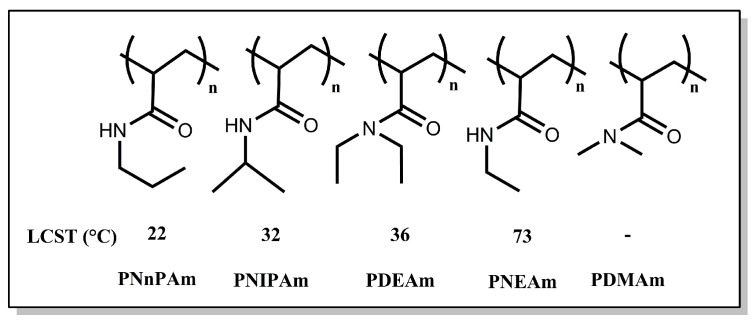
Some *N*-alkyl-substituted polyacrylamides and their respective LCSTs: PNnPAm: poly(*N*-n-propylacrylamide), PNIPAm: poly(*N*-isopropylacrylamide), PDEAm: poly(*N,N*-diethylacrylamide), PNEAm: poly(*N*-ethylacrylamide), PDMAm: poly(*N,N*-dimethylacrylamide) [23].

Some *N*-alkyl-substituted polyacrylamides show a thermal phase transition in an aqueous solution characterized by a lower critical solution temperature (LCST). As shown in Figure 1, there is a wide variation in thermoresponsive properties within this polymer family. As both poly(*N*-ethylacrylamide) (PNEAm) and poly(*N,N*-diethylacrylamide) (PDEAm) experience a coil-to-globule transition in aqueous solution, the LCST of the former is much larger than that of the latter [23,24]. In contrast, poly(*N,N*-dimethylacrylamide) shows no thermoresponsive behavior in aqueous solutions. The occurrence of the coil-to-globule transition can be understood as a balance between hydrophobic and hydrophilic interactions in polymer chains with temperature [25]. Below the LCST, the *N*-substituents in the acrylamide groups are hydrated as well as the amide group, which results in the formation of so-called *icebergs* around the amide groups through hydrogen bonding interactions with water molecules. This maintains the polymer chains in a hydrated coil state. An increase in temperature promotes hydrophobic interactions along the polymer chain, causing it to shrink to a hydrophobic globule state [23]. When hydrophobic *N*-substituents are exposed on the surface of the globules, they promote the globule aggregation processes. Hydrogen bonding interactions can also occur within the same chain, between neighboring chains, or with residual water molecules.

The LCST depends on factors associated with the macromolecular architecture of the polyacrylamides such as the molecular weight [26,27,28], the terminal group of the polymer chain [28], or the type of *N*-alkyl substituents [29], among others [24,30]. For example, it can be mentioned that the presence of the –NH group of the secondary amide improves hydrogen bonding interactions with water, stabilizing the hydrated form of the polymer and increasing the LCST. On the other hand, it is also known that the addition of surfactants or chaotropic substances to polyacrylamide solutions can abruptly change the nature and the spatial ordering of long-range interactions, generating important changes in the value of the cloud-point temperature, and the phase transition itself [27,31,32,33].

Little is yet known about the role of the secondary or tertiary amide group in modulating the phase transition temperature, and the interactions that prevail and determine these changes [3,34,35,36]. With the aim of understanding their role in the phase transition, this study focuses on the molecular interactions underlying the phase transition of some poly(*N*-alkyl acrylamides), particularly in aqueous solutions of poly(*N,N*-diethylacrylamide-*co*-*N*-ethylacrylamide) random copolymers and their respective homopolymers. The influence of a chaotropic agent on the thermal transition of aqueous solutions of these materials was also investigated.

## 2. Materials and Methods

### 2.1. Materials

*N,N*-diethylacrylamide (DEAm) (Sigma-Aldrich, St. Louis, MO, USA, 99.0%) and *N*-ethylacrylamide (NEAm) (Sigma-Aldrich, 99.0%), 2-propanol (Sigma-Aldrich, 99.5%), acetone (Fermont, Monterrey, NL, Mexico, 99.7%), n-hexane (J.T. Baker, Phillipsburg, NJ, USA, 98.0%), diethyl ether (Faga Lab, Mocorito, Mexico, 99.8%), chloroform (Sigma-Aldrich, 99%), and urea (Sigma-Aldrich, 99%) were used as received. The initiator 4,4′-azobis(4-cyanovaleric) acid (ACVA) (Sigma-Aldrich, 98.0%) was purified by recrystallization with ethanol. Type I ultrapure water with a conductivity lower than 0.05 μS cm^−1^ was used in all experiments.

### 2.2. Synthesis of PDEAm, PNEAm Homopolymers, and PDEAm-co-PNEAm Random Copolymers by Free Radical Polymerization

The homopolymers PDEAm and PNEAm and random copolymers (A, B, and C) with different compositions were synthesized by free radical polymerization [37,38]. In each case, the initiator ACVA and the corresponding monomer (or their combination) were dissolved in 2-propanol and added to a sealed vial (details in Table 1 and Scheme S1). Oxygen was removed by bubbling nitrogen into the vial for 30 min. The vial was then placed in a preheated bath at 65 °C and allowed to polymerize for 24 h. The resulting polymer was precipitated in cold n-hexane and separated by centrifugation (T = 10 °C, 4200 rpm for 40 min). After decanting, the polymer was redissolved in acetone, and this cycle was repeated three times. The precipitate was dried under vacuum at room temperature, redissolved in water, and dialyzed using Spectra/Por^®^ membranes (MWCO = 1000) until the ionic conductivity reached that of pure water and then lyophilized.

### 2.3. Size Exclusion Chromatography (SEC-MALS)

Polymer samples were dissolved in type I water (1 mg mL^−1^) and filtered through cellulose membranes (0.45 µm) (Sartorius Biotech GmbH, Goettingen, Germany). An AF2000 multiflow system from Postnova Analytics (Postnova Analytics GmbH, Landsberg, Germany), was operated in SEC mode and coupled with a multi-angle light scattering detector (21 angles, PN3621), a dual UV detector (l = 220 and 280 nm, PN3211) and a refractive index detector (PN3250). Two columns OHpak SB-802.5 HQ (exclusion limit: 1 × 10^4^ g mol^−1^) and SB-806 M HQ (exclusion limit 2 × 10^7^ g mol^−1^) (Showa Denko Europe GmbH, Munich, Germany) were used and the temperature was controlled using a thermostat (PN4020) at 30 °C. Analysis of the data was performed using NovaMALS (Postnova Analytics GmbH, ver. 1.5.0.7). The eluent was type I water filtered through a membrane (0.1 µm), the dn/dc value used in all cases was 0.1627 [39]. All measurements were made in duplicate.

### 2.4. Turbidity Measurements

Cloud-point temperature was determined by thermo-optical measurements using a Shimadzu BioSpec-1601 spectrophotometer equipped with a manually operated Shimadzu CPS-240A temperature control device. Transmittance was measured at λ = 500 nm after allowing at least 3 min for reaching thermal equilibrium.

With this purpose, aqueous polymer solutions were prepared and left to stand for equilibrium at 4 °C for at least 48 h. Preliminary experiments showed that the transition is well defined at concentrations 2 mg mL^−1^ or higher, but it becomes diffuse below this concentration. Thus, all polymers were dissolved in water (2 mg mL^−1^) and placed into quartz cuvettes for measurements.

### 2.5. Dynamic Light Scattering (DLS) and ζ-Potential

Hydrodynamic radius and ζ-potential measurements were performed in a temperature-controlled Möbiuζ instrument (Wyatt Technology, Santa Barbara, CA, USA). Unless otherwise stated, all samples were dissolved in water (2 mg mL^−1^) following the same protocol described before for turbidity measurements. These solutions were filtered through a membrane (0.45 μm) when loaded into a 45 μL quartz cuvette with electrode assembly for DLS and ζ-potential measurements. A heating program (0.6 °C min^−1^, 25–68 °C) was used during analyses. Once the measurement temperatures were reached, the sample was thermally equilibrated for 10 min within ±0.05 °C. 

The Möbiuζ system uses a 45 mW single longitudinal-mode laser operating at a wavelength of 532 nm. The measurements were made at a scattering angle of θ = 163.5°. Data acquisition and processing—including the estimation of the molecular weight—were performed using Dynamics 7.10.1.21 software (Wyatt Technology, Santa Barbara, CA, USA).

### 2.6. Fourier Transform Infrared Spectroscopy (FTIR)

FTIR spectra were collected on a Nicolet iS-50 (Madison, WI, USA) with an attenuated total reflectance (ATR) module, using a total of 64 scans with a resolution of 2 cm^−1^.

### 2.7. Proton Nuclear Magnetic Resonance Spectroscopy (^1^H-NMR)

The spectra were recorded on a Bruker Avance 400 (400 MHz) spectrometer at 25 °C. Polymer samples were dissolved (12 mg mL^−1^) in deuterated chloroform (CDCl_3_) using tetramethylsilane (TMS) as an internal reference.

## 3. Results and Discussion

### 3.1. Polymer Synthesis and Structural Characterization

PDEAm and PNEAm homopolymers and PDEAm-*co*-PNEAm random copolymers were obtained by free radical polymerization using a similar procedure as that reported by Fernández-Quiroz et al. [37]. In all cases, the same synthetic procedure described above was followed, and white powder lyophilizates were obtained. All samples were readily soluble in water and chloroform.

In Table 1, the nominal feed composition of the three random copolymers (A, B, C with increasing DEAm content) is presented, as well as the percentage yields obtained for the five samples. The latter varied between 55 and 77%, which agrees with the values reported for this type of polymerization [37,39,40].

**Figure 2:**
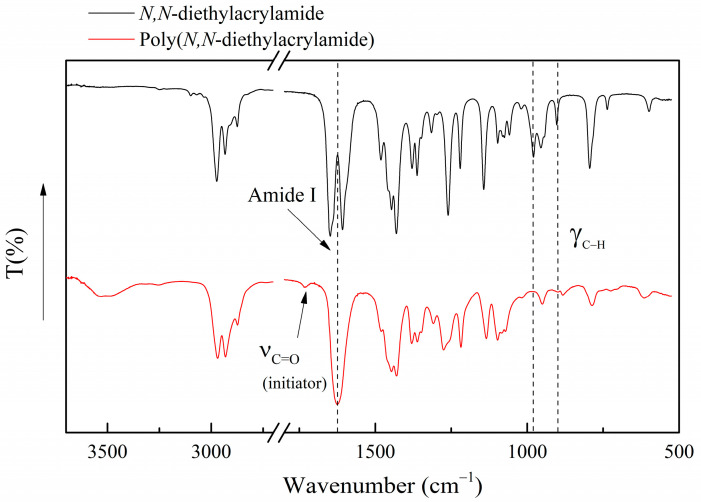
ATR Fourier-transform infrared spectra of *N,N*-diethylacrylamide and poly(*N,N*-diethylacrylamide).

Figure 2 shows the infrared spectra of *N,N*-diethylacrylamide and its homopolymer (PDEAm). The main differences between the spectra of the monomer and the homopolymer are evident. A couple of bands at 1610 and 1650 cm^−1^ appear in the monomer spectrum, corresponding to the coupling between the vibrations of the vinyl (ν_C = C_) and carbonyl groups (ν_C = O_). However, at 1620 cm^−1^, in the spectrum of PDEAm, only an intense band (ν_C = O_), corresponding to the vibrations of the carbonyl group (amide I band), is observed. This change is consistent with the disappearance of vinyl groups due to the polymer formation during the radical polymerization reaction. Out-of-plane bending vibrations of the C–H bonds of the monosubstituted unsaturated vinyl group are found at 980 cm^−1^ and 900 cm^−1^, which also disappear in the spectrum of the polymer. This indicates that polymerization has indeed occurred through the vinyl groups of the monomers, and there is no evidence of residual monomer in the sample. It is worth noting an incipient signal at 1732 cm^−1^ in the PDEAm corresponding to the carboxylic group of the initiator residue at the end of the polymer chain, which is not present in the spectrum of the monomer. 

Both samples (monomer and the corresponding homopolymer) share some other characteristic bands, which is consistent with the radical polymerization process. In this sense, it could be mentioned the bands associated with bending vibrations of CH_2_ and CH_3_ groups at 1480–1430 cm^−1^. Additionally, in the 1300–1100 cm^−1^ region, the absorptions corresponding to the bending of the C–CO–C group (1264 cm^−1^) and in-plane bending (rocking) of the CH groups (1160 cm^−1^) could be noted.

**Figure 3:**
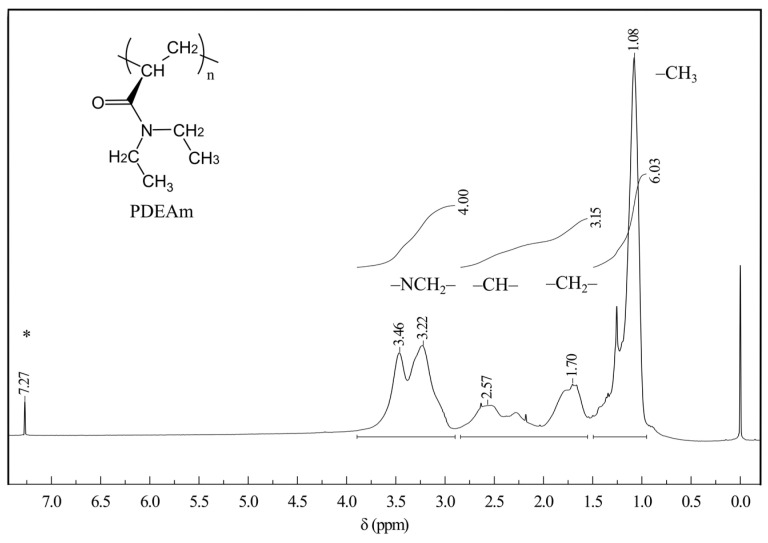
Proton nuclear magnetic resonance spectrum of poly(*N,N*-diethylacrylamide) dissolved in CDCl_3_. The integration of the signals is also included. The asterisk denotes residual solvent signal.

The ^1^H-NMR spectrum of poly(*N,N*-diethylacrylamide) is presented in Figure 3. The signal of the methyl group protons (–C**H**_3_) at 1.08 ppm and those of the methylene groups linked to the nitrogen (–NC**H**_2_–) at 3.46 and 3.22 ppm can be appreciated. The presence of two signals for the latter has been explained as a consequence of the magnetic anisotropy exerted by the carbonyl group [41]. The protons of the –C**H**_2_– and –C**H**– groups of the polymer backbone appear at 1.70 and 2.57 ppm, respectively [42]. Note that there is no signal in the region between 5 and 7 ppm, where protons bonded to unsaturated carbons should resonate. This confirms the result obtained from the infrared spectrum. The integration of the signals is also included in Figure 3. A ratio of 3:4:6 is evident, corresponding to the relation of the principal protons of the polymer, which unequivocally corroborates the structure of the polymer.

**Figure 4:**
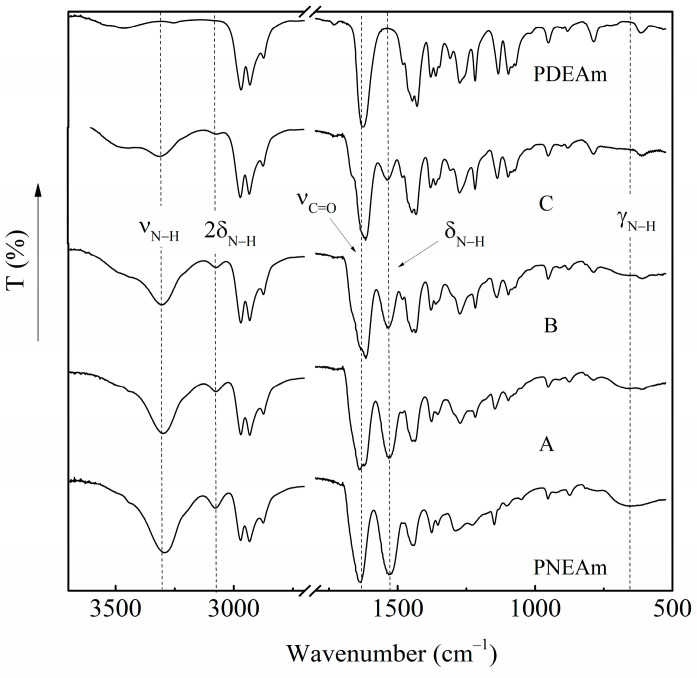
ATR Fourier-transform infrared spectra of the PDEAm and PNEAm homopolymers and A, B, and C copolymers.

Figure 4 shows the FTIR spectra of the five polymer samples obtained by free radical polymerization. As expected, the presence of both *N*-ethylacrylamide and *N,N*-diethylacrylamide structural units in the copolymer samples is evident. The amide I band (ν_C = O_), associated with the vibrations of the carbonyl groups, appears at 1650 cm^−1^ and does not show significant variations. The amide II band (δ_N–H_) related to the in-plane bending of the N–H bonds arises at 1530 cm^−1^. The latter could be used as an indicator of the NEAm content in the copolymer samples, as it is only present in NEAm units. It is noteworthy that the intensity of the amide II band increases considerably as the *N*-ethylacrylamide content in the copolymers increases, exhibiting a maximum intensity for PNEAm. It is absent from the PDEAm spectrum. Something similar happens with the out-of-plane folding band of the N–H bonds (γ_N–H_) that appears at 655 cm^−1^. It can be seen as a broad and weak band associated with the presence of *N*-ethylacrylamide units.

**Figure 5:**
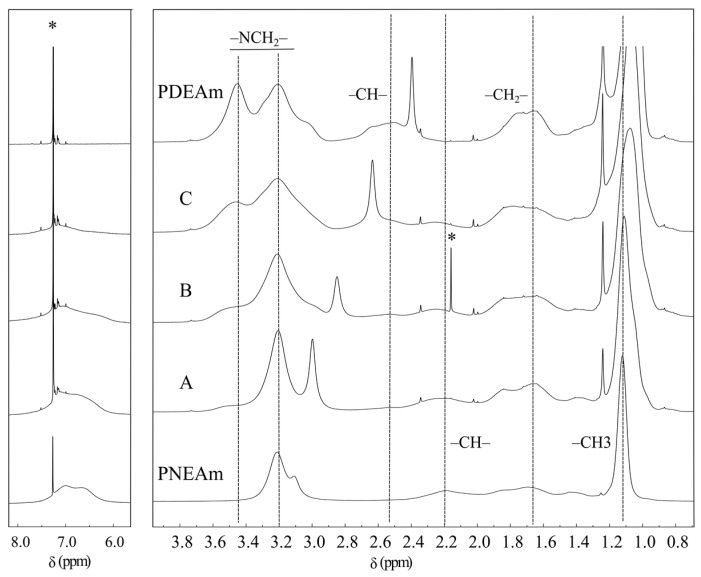
Proton nuclear magnetic resonance spectra of the PDEAm and PNEAm homopolymers and A, B, and C copolymers dissolved in CDCl_3_. The asterisks denote residual solvent signal.

In Figure 5, the ^1^H-NMR spectra of the copolymers and both homopolymers are shown. Signals from methylene groups linked to the nitrogen (–NC**H**_2_–) in the PDEAm spectrum can be observed at 3.20 and 3.45 ppm, whereas in the PNEAm spectrum, only one signal appears at 3.20 ppm. In the copolymers, the intensity of the signal at 3.45 ppm steadily increases as the content of *N,N*-diethylacrylamide units increases. The proton signal of the –C**H**– groups of the main chain appears in the PDEAm spectrum at 2.55 ppm as a broadened and weak band. In PNEAm, this band presents similar characteristics but is somewhat more shielded at 2.2 ppm. The peak of methylene groups (–C**H**_2_–) of the polymer backbone is located at 1.7 ppm, while the signal of the methyl groups (–C**H**_3_) is found at 1.1 ppm. As observed in Figure 3, there is also good correspondence in the relation of integration of the main protons of the polymer.

From the proton NMR spectra, it was possible to calculate the molar composition of the copolymers from the integral of the signal of the –C**H**_3_ protons at 1.1 ppm with respect to that of the three protons of the vinyl chain (–C**H**– and –C**H**_2_– signals, Figure 5). The experimental molar fractions of both monomers used during polymerization and the molar fraction composition calculated for the three copolymers from NMR spectra are summarized in Table 2. Except for the composition of copolymer B, the molar fraction composition values agree acceptably with those of the reactant mixture. The observed differences might be related to the dissimilar reactivity ratios between both monomers and agree with findings reported by other authors [24,43].

Several attempts were made to measure the molecular weight and polydispersion of the five samples by SEC-MALS-DRI, but these were only partially successful. These results were particularly critical for the PDEAm and copolymer C samples, where only 6 and 18% recovery were obtained, respectively. This is most likely due to phase separation phenomena because the temperature in the instrument was unable to be consistently controlled below the cloud point. 

A rough estimation of the molecular weight by dynamic light scattering at 25 °C was performed as well. For this purpose, calculations were performed assuming that the polymer chains were in a random coil conformation at 25 °C, a temperature well below the cloud-point temperature of these samples. Moreover, it must be considered that these polyacrylamide samples are neutral, so no coulombic interactions could affect their random coil configuration in dilute solutions. This is a perfectly reasonable assumption for a dilute aqueous solution of these polymers at the indicated temperature. 

The weight-average molecular weights—obtained by DLS and SEC-MALS-DRI—the polydispersity index, and the corresponding degree of polymerization are summarized in Table 3. It is worth mentioning that the molecular weight values of PNEAm and copolymers A and B—which are the samples with the highest cloud-point temperatures—obtained by both methods, compare very satisfactorily. By SEC, the measured values for these three samples were 19, 31, and 25 kDa, respectively (samples with mass recovery percentages higher than 88%). Samples C and PDEAm had very low mass recoveries of 17.7 and 5.5%, respectively, which did not allow any meaningful calculation of the molecular weight parameters to be made. Regarding the PDI values, it can be appreciated that values between 1.3 and 1.6 were obtained, which are very satisfactory. These values are in good agreement with those reported for polyacrylamides synthesized in protic solvents under RAFT conditions (values between 1.2 and 1.4) [44].

The good agreement between the molecular weight estimated by DLS and the results obtained by SEC for the samples with higher cloud-point temperatures gives greater certainty to the data obtained by DLS. Nevertheless, it should be recalled that DLS is not an absolute method for measuring molecular weight. It could provide a rough estimation of the molecular weight of single macromolecules via the use of a linear dependence between the molecular weight and the hydrodynamic size for certain samples under very specific conditions. 

It could be appreciated that the degree of polymerization ranged between 200 and 260, thus confirming that the five polymer samples have similar average lengths. Such DP values are quite similar to those obtained for poly(*N*-vinyl caprolactam) synthesized under similar conditions [37]. They also compare with PDEAm homopolymer [45], and PNEAm and its copolymers with poly(*N*-isopropyl acrylamide) obtained under usual radical polymerization conditions [24].

### 3.2. Analysis of the Phase Transition in Aqueous Solution

With the aim of understanding and gaining knowledge on the interactions involved in the phase transition of copolymers of these two *N*-alkyl-substituted units, the thermal transition of their aqueous solutions was studied by means of turbidimetry, dynamic light scattering, and ζ-potential. Unfortunately, it was not possible to analyze the behavior of PNEAm homopolymer solutions since their thermal transition occurs at temperatures higher than those achievable with the available instruments. Therefore, for this specific sample, the cloud-point temperature was measured using the traditional visual method—which is not the most accurate—giving a value of 80 °C. This figure is in close agreement with the previously reported data for a series of PNEAm samples of different molecular weights dissolved in water, taking into account the molecular weight of the sample used in this work (20 kDa, Table 3) [27].

**Figure 6:**
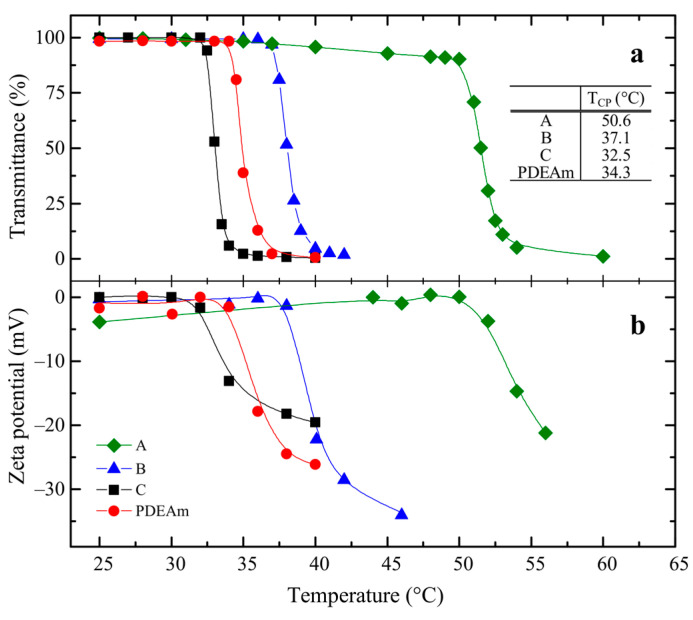
Temperature dependence of (**a**) transmittance, and (**b**) ζ-potential of aqueous solutions of PDEAm and A, B, and C random copolymers. Polymer concentration: 2 mg mL^−1^.

The traces of turbidimetric measurements (shown in Figure 6a) show the typical pattern of the thermal transition during phase separation of polymer solutions, evidenced by an abrupt drop-off in transmittance for the copolymers and PDEAm solutions with temperature. The transition is observed when the cloud-point temperature is reached, pointing to the development of the transition. Since the cloud-point temperature, T_CP_, is defined as the temperature at which an incipient opalescence takes place due to the phase separation phenomena, it was estimated from the intersection of the tangents of the transmittance curve before and just after the onset of turbidity.

There is an overall rise in the cloud-point temperature of the copolymers with increasing NEAm content in the copolymer (Table inside Figure 6a). The NEAm units confer more hydrophilic properties to the copolymer due to the presence of NH groups in its structure, thus promoting hydrogen bonding between copolymer chains and water. Nevertheless, at the same time, one can notice that the cloud-point temperature of copolymer C is not higher than that of the poly(*N,N*-diethylacrylamide) solution, but unexpectedly almost 2 °C lower than the latter.

**Figure 7:**
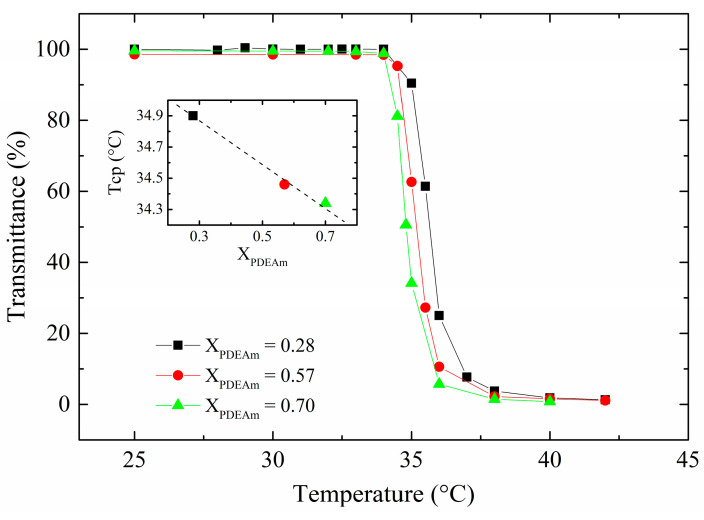
Turbidity measurements of solutions of mixtures of the homopolymers (PDEAm and PNEAm) at the corresponding molar fraction. Polymer concentration: 2 mg mL^−1^. Inset: Cloud-point temperature (T_CP_) dependence with the PDEAm molar fraction in the homopolymer mixtures.

To shed light on this behavior, solutions of physical mixtures of PDEAm and PNEAm homopolymers at the same molar fractions of the random copolymers were also analyzed. From Figure 7, it is evident that the cloud-point temperatures of the solutions of the homopolymer mixtures are very close to each other and almost equal to that of the pure PDEAm solution. There is only a very slight tendency for the cloud-point temperature to increase as the PNEAm content in the homopolymer blend becomes higher (see inset in Figure 7). Under these conditions, the thermal transition of PDEAm chains is virtually not affected by the presence of PNEAm chains in the same solution, the latter remaining soluble with essentially no interference between both homopolymer chains. The observed behavior is very clarifying about the role played by the interactions appearing between both structural units when they are forced close to each other within the macromolecular coil of a copolymer. This contrasts with the absence of interactions observed when they both are part of their respective homopolymers, even when the homopolymers are mixed in the same solution.

**Figure 8:**
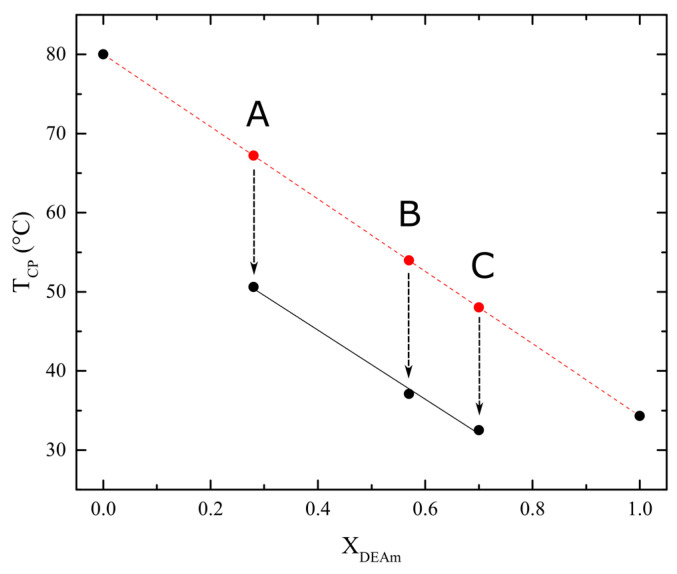
Cloud-point temperatures (T_CP_) of the synthesized polymers as a function of their molar fraction composition. Red symbols are the calculated values for the A, B, and C copolymers in the absence of interactions between their structural units, as calculated from Equation (1). Black symbols are the experimentally measured T_CP_ for the two homopolymers and copolymers.

To understand this overall behavior, Figure 8 depicts the data of the measured cloud-point temperatures as a function of the molar fraction composition of the three copolymers, X_DEAm_ (black points). The experimental values of the homopolymers are included as well (black points). On the other hand, the hypothetical value of the cloud-point temperature of each copolymer was calculated assuming a simple linear dependence between their molar copolymer composition and the experimental *T_CP_* values of both homopolymers, in a similar dependence as that used before for statistical copolymers of *N*-isopropylacrylamide and *N*-isopropylmethacrylamide [46].

(1) $${{Tcp}_{copolym.theoretical}=Tcp}_{PNEAm}-X_{DEAm}\left({Tcp}_{PNEAm}-{Tcp}_{PDEAm}\right)$$

where X_DEAm_ is the molar fraction of diethylacrylamide of the copolymer (shown in Table 2), and *Tcp_PNEAm_* and *Tcp_PDEAm_* are the cloud-point temperatures of PNEAm and PDEAm homopolymers, respectively.

These theoretical values are also displayed in Figure 8 (red points). From a physical point of view, these calculated cloud-point temperatures can be considered as those that would be expected for the copolymers in the absence of intra- or interchain interactions between both structural units.

**Figure 9:**
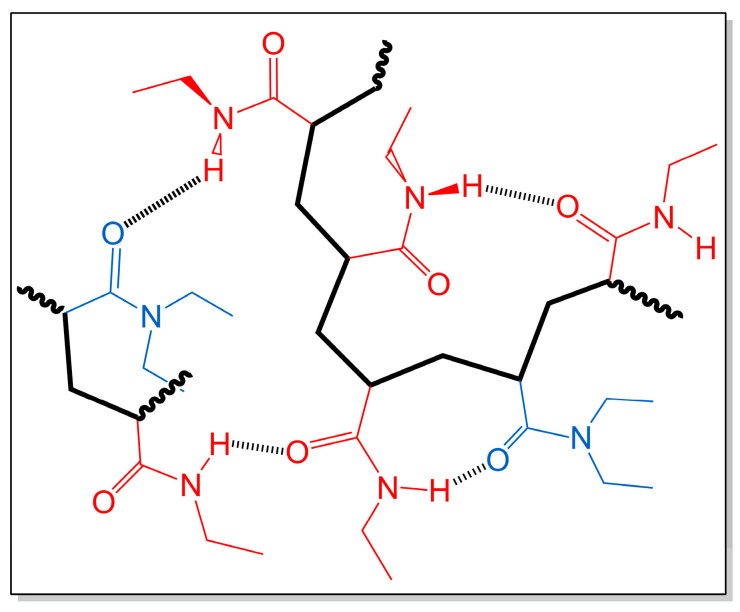
Possible intra- and interchain hydrogen bonds in aqueous solutions of poly(*N,N*-diethylacrylamide-*co-N*-ethylacrylamide) random copolymers.

There are reports in the literature about different parameters that can influence the T_CP_ of a thermosensitive polymer: molecular weight and its distribution [26,27,28], polymer concentration [26], presence of ionic monomeric units [47,48], stereoregularity, and tacticity [24,30], among others. In this case, it is evident that there is a remarkable decrease in the T_CP_ of each copolymer with respect to the theoretical value assuming no interactions inside the random coils of the copolymers. Normally, the presence of a certain amount of more hydrophilic units of *N*-ethylacrylamide to the *N,N*-diethylacrylamide chain would imply a more hydrophilic behavior of these macromolecules in solution and, therefore, an increase in the transition temperature [49,50]. However, this is not the case. Thus, the observed decrease in T_CP_ can only be understood by the presence of hydrogen bonding between both types of units, as depicted in Figure 9. In this case, it can be suggested that the appearance of intra- and interchain hydrogen bonds in the aqueous solutions of the copolymers may (*i*) favor the formation of more hydrophobic macromolecular segmental zones and (*ii*) favor the cooperative character of the coil-to-globule transition due to the forced proximity between those two acrylamide units inside the copolymer macromolecular coils. In both cases, it is expected that the presence of more hydrophilic *N*-ethylacrylamide units, instead of increasing the cloud-point temperature, will have the opposite effect. The breaking of hydrogen bonds between acrylamide units and water results in the formation of hydrogen bonds between monomer units. This phenomenon is most likely to lead to an increase in hydrophobic interactions within the macromolecular domain. A similar conclusion has also been described in other studies with hydrogels containing PNIPAm and PDEAm (secondary and tertiary acrylamide groups, respectively) [34,35]. 

Figure 6b summarizes the ζ-potential measurements as a function of temperature for the studied polymer solutions. In general, there is a good coincidence between the dependence of the transmittance and the ζ-potential with temperature (Figure 6a,b). The ζ-potential is a parameter related to the surface charge of the particles dispersed in an aqueous medium. In this case, it shows values close to 0 mV for temperatures below the corresponding LCST, suggesting that these macromolecular samples have almost zero surface charge due to the well-solvated neutral macromolecular coils in solution. Above the cloud-point temperatures, with the onset of the coil-to-globule transition, negative ζ-potential values appear, which gradually increase as polymeric aggregates progressively form. 

The appearance and increase in the negative surface charge with the process of the coil-to-globule transition may at first sight seem strange and contradictory, especially since it is a neutral polymer. However, this is a phenomenon that has been observed and previously investigated, occurring during the phase transition of thermosensitive microgel particles such as PNIPAm in water [51,52]. It has been suggested that adsorption of OH^−^ ions happens at the water/hydrophobic interface, causing negative electrophoretic mobility values [53]. Theoretical molecular dynamics simulation studies have confirmed this hypothesis and suggest that it is a spontaneous process. From a physical point of view, it seems to be due to the orientation of the water molecules in the two inner hydrophobic solvation layers and due to long-range effects on the electric potential gradient [54]. The same phenomenon has been found to occur as a result of phase transition in thermosensitive polymers regardless of the ionic nature of the radical initiator used [38,51]. However, the specific effect of the carboxylic groups from the residues of the initiator at the end of the polymer chains cannot be ruled out either.

**Figure 10:**
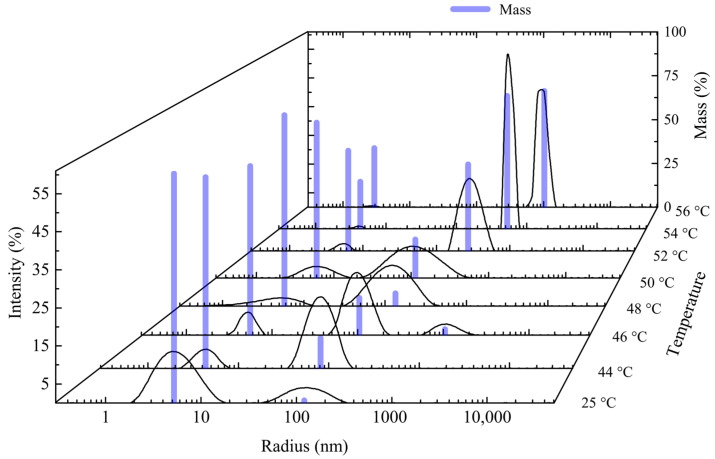
Intensity of light scattered (black traces) and corresponding mass fraction (blue bars) as a function of the hydrodynamic radius at different temperatures. Copolymer A dissolved in water. Polymer concentration: 2 mg mL^−1^.

The intensity of scattered light as a function of the hydrodynamic radius is presented in Figure 10, together with their corresponding mass fraction measured at different temperatures in an aqueous solution of copolymer A. From 25 to 50 °C, most of the polymer mass (>90%) is in the solvated state, with a hydrodynamic radius below 10 nm. Above 50 °C, the mass fraction of particles with larger hydrodynamic radius starts to increase, suggesting that the thermal transition has occurred and, consequently, the aggregation of the polymer particles takes place. A similar behavior was found for PDEAm and the other two copolymer samples, but at different temperatures (Figures S1–S3).

Chaotropic agents are substances capable of disturbing the spatial arrangement of macromolecules in aqueous solutions since they interfere with the hydrogen bonding network of water, leading to changes in the conformation of macromolecules and destabilizing the structure of the hydration *icebergs* around the hydrophobic groups of the macromolecule. Urea is a well-known chaotropic agent capable of interacting with polymer chains by exchanging the water molecules of the solvation sphere associated with the amide groups [31].

It has been found that the structure of the amide group has a great influence on the thermal behavior of polyacrylamide aqueous solutions in the presence of urea. In the case of PDEAm, an increase in LCST was observed with a higher urea concentration, which is related to the stabilization of the coil conformation [32]. On the other hand, urea contributes to stabilizing the globular conformation of poly(*N*-isopropylacrylamide), leading to a decrease in its LCST [31,33]. Wang et al. explained this by suggesting that urea could not only interact directly with PNIPAm via hydrogen bonding but also promote hydrogen bonding interactions between water and the polymer chain. The presence of the amide hydrogen plays a key role in this mechanism [32]. From a theoretical approach, Pica and Graziano concluded that this different behavior observed in PDEAm and PNIPAm could be due to urea interacting not only with the amide group through hydrogen bond interactions but also with the alkyl substitutions through van der Waals interactions. The *N*-ethyl groups in PDEAm offer a larger surface area to interact with urea molecules when compared to the *N*-isopropyl groups in PNIPAm, thus favoring the stabilization of the coil conformation in PDEAm [55]. In the case of PNEAm, a similar behavior to that of poly(*N*-isopropylacrylamide) could be expected since both polymers have secondary amide groups in their structure.

**Figure 11:**
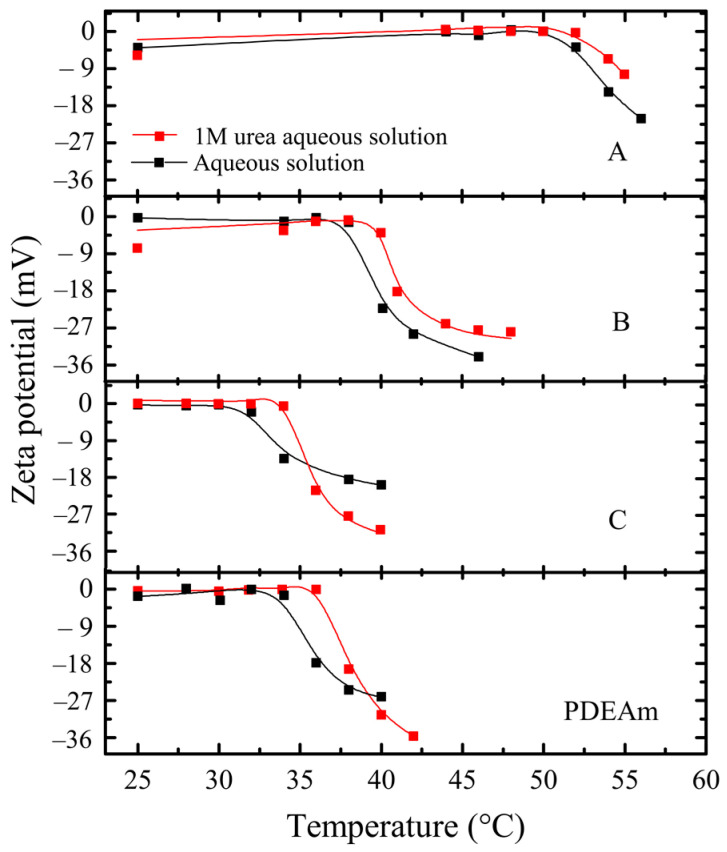
ζ-potential measurements as a function of temperature of PDEAm and A, B, and C random copolymers dissolved in water (black symbols and curves) and 1 M urea (red symbols and curves). Polymer concentration: 2 mg mL^−1^.

Figure 11 shows the dependence of ζ-potential on the temperature of PDEAm, and A, B, and C random copolymers dissolved in water and 1 M urea. An increase in the LCST of all four polymers (approximately 2 °C) is observed in the presence of urea, even in those copolymers with a higher proportion of *N*-ethylacrylamide units. Like the behavior exhibited by the aqueous solutions of these polymers, an increase in the negative value of the ζ-potential of the aggregates is also observed in the presence of the chaotropic agent at temperatures above the LCST.

It can be concluded that although urea seems to stabilize the macromolecular globule of these polymers regardless of the composition of both *N*-alkylacrylamide groups (an increase in the cloud-point temperature ≈ 2 °C), the coil-to-globule transition and surface charge of the globules are comparable to those obtained in their aqueous solutions.

The findings of this study help to understand the role of the interactions between the functional groups of these polyacrylamides in their thermosensitive properties. Further investigation indicates that it is possible to graft these *N*-alkyl-substituted polyacrylamides with a functionalized chain end onto chitosan and prepare pH- and temperature-sensitive polymeric materials [37,56]. This has a positive impact on the design of materials with enhanced properties for biotechnological and biomedical applications, with their full potential yet to be realized [11].

## 4. Conclusions

The phase transition of poly(*N,N*-diethylacrylamide-*co*-*N*-ethylacrylamide) random copolymers in aqueous solution was characterized by cloud-point temperature, hydrodynamic radius, and ζ-potential measurements. In this regard, the cloud-point temperature increased with increasing NEAm content in the copolymers. At the same time, a particle aggregation process takes place during the transition, thus increasing the negative charge density on their surface. 

A remarkable decrease in the values of cloud-point temperatures was observed with respect to the theoretical values calculated assuming an absence of interactions. By increasing the content of more hydrophilic NEAm units in the copolymers, the formation of intra- and interchain hydrogen bonds between both types of acrylamide groups is favored. These interactions result in the formation of more hydrophobic macromolecular segments, thereby promoting the cooperative nature of the coil-to-globule transition process due to the forced proximity between acrylamide units along the polymer chain. The presence of urea stabilizes the hydrated state of the chains, increasing the LCST of the copolymers. These results definitively reveal the dominant mechanism occurring during the phase transition in the aqueous solutions of these copolymers. This approach enables us to understand the underlying structure–property relationships and the ability to design advanced materials with targeted properties.

## Figures and Tables

**Table 1:** Quantities and yields achieved in polymers synthesized by free radical polymerization.

Sample	X (M_1_) ^a^	m (M_1_) (g)	m (M_2_) (g)	Rm (mol-%) ^b^	Yield (%)
PNEAm	0.00	0.0000	1.779	0.71	77.1
A	0.21	0.7502	2.2546	0.74	65.2
B	0.44	1.5048	1.5136	0.79	77.2
C	0.70	2.2528	0.7502	0.85	54.8
PDEAm	1.00	2.9982	0.0000	0.89	73.6

^a^ X: Molar fraction of M_1_; ^b^ R_m_: Ratio of moles of initiator per 100 moles of monomer; M_1_: *N,N*-diethylacrylamide, M_2_: *N*-ethylacrylamide.

**Table 2:** Molar fraction composition of the comonomer units on the reaction mixture, and the calculated values for the synthesized copolymers.

Sample	X_DEAm_ ^R^	X_DEAm_ ^C^
PNEAm	0.00	0.00
A	0.21	0.28
B	0.44	0.57
C	0.70	0.70
PDEAm	1.00	1.00

^R^ Molar fraction composition of the reaction mixture. ^C^ Molar fraction composition of the synthesized copolymers as calculated by ^1^H-NMR.

**Table 3:** Weight-average molecular weights, degree of polymerization, and molecular weight polydispersity index of the polymer samples and the mass recovery percentages, estimated from DLS and SEC-MALS-DRI measurements.

	DLS Mw (kDa)	DP *	SEC Mass Recovery (%)	SEC Mw (kDa)	SEC Mw/Mn Polydispersity Index
PNEAm	20.0	201	91.7	19.3	1.58
A	28.0	261	90.2	30.8	1.38
B	26.6	231	88.5	25.4	1.29
C	31.2	263	17.7	—	—
PDEAm	24.7	194	5.5	—	—

* The DP values were calculated considering the corresponding molar fraction of both units for each copolymer.

## Data Availability

Data are contained within the article.

## Supplementary Materials

The following supporting information can be downloaded at: https://www.mdpi.com/article/10.3390/polym16111575/s1, Scheme S1: Schematic diagram describing the polymer synthesis processes. Figures S1–S3: Intensity of light scattered and corresponding mass fraction as a function of the hydrodynamic radius at different temperatures. PDEAm and copolymers B and C dissolved in water.
