# Supplementary material for: Study of the Thermal Phase Transition of Poly(N,N-diethylacrylamide-co-N-ethylacrylamide) Random Copolymers in Aqueous Solution
Source: Polymers (Basel). 2024 Jun 2;16(11):1575. doi: 10.3390/polym16111575 (PMC11175111; doi:10.3390/polym16111575)
Supplement: Supplementary file 1 [file polymers-16-01575-s001.zip › polymers-2944019-supplementary.pdf]

Supplementary Material

Study of the thermal phase transition of poly(*N,N*-diethylacrylamide-*co*-*N*-ethylacrylamide) random copolymers in aqueous solution

José Javier Coca-Hidalgo [1], Maricarmen Recillas-Mota [1], Daniel Fernández-Quiroz [2], Jaime Lizardi-Mendoza [1], Carlos Peniche-Covas [3], Francisco M. Goycoolea [4,†] and Waldo M. Argüelles-Monal [1,*]

[1] Centro de Investigación en Alimentación y Desarrollo, 83304 Hermosillo, Sonora, Mexico; jcoca221@estudiantes.ciad.mx (J.J.C.-H.); mrecillas@ciad.mx (M.R.-M.); jalim@ciad.mx (J.L.-M.)
[2] Departamento de Ingeniería Química y Metalurgia, Universidad de Sonora, Hermosillo, Mexico; daniel.fernandez@unison.mx
[3] Facultad de Química, Universidad de La Habana, La Habana, Cuba; cpeniche2015@yahoo.com
[4] School of Food Science and Nutrition, University of Leeds, LS2 9JT Leeds, U.K.
[*] Correspondence: waldo@ciad.mx
[†] Present address: Facultad de Biología, Universidad de Murcia, 30100 Murcia, Spain; fmartin.goycoolea@um.es

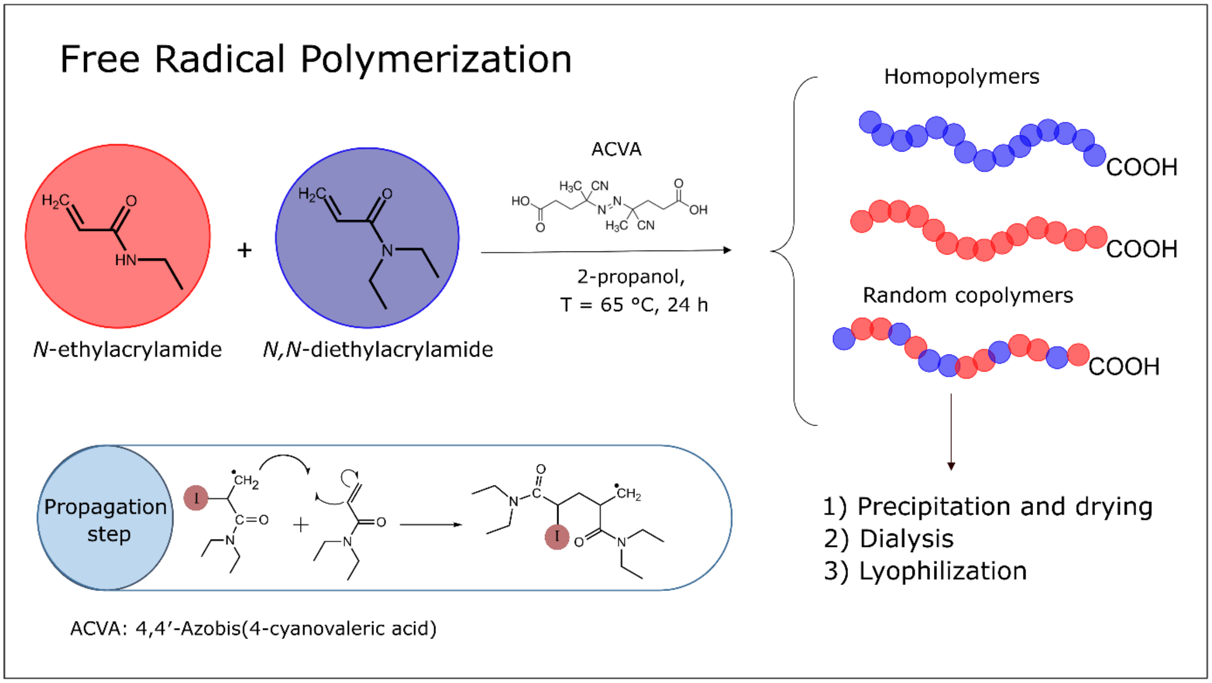

Scheme S1. Schematic diagram describing the polymer synthesis processes.

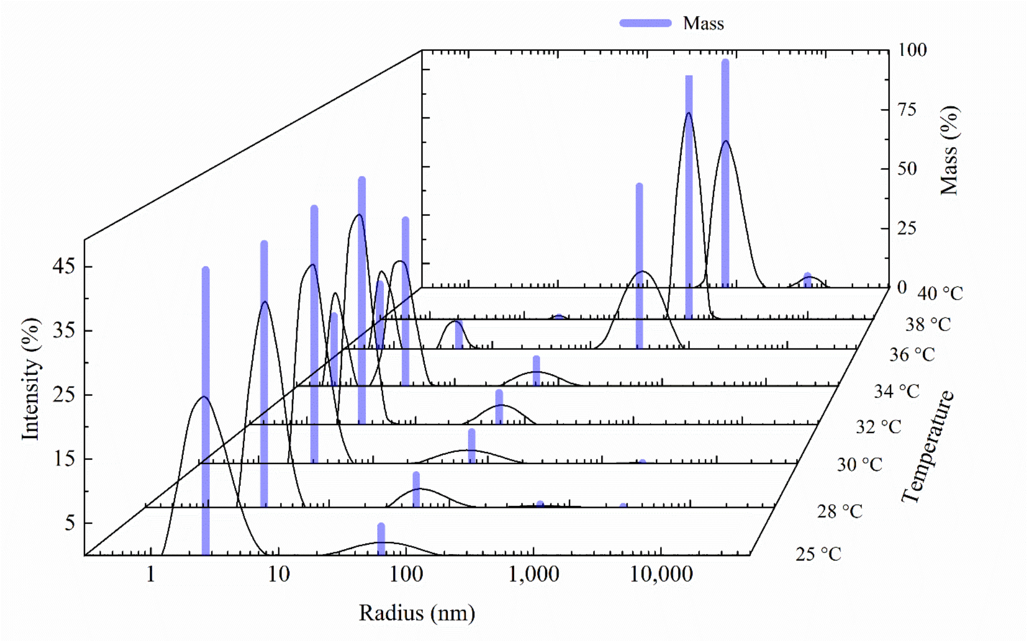

Figure S1. Intensity of light scattered (black traces) and corresponding mass fraction (blue bars) as a function of the hydrodynamic radius at different temperatures. PDEAm dissolved in water (2 mg mL^{-1}).

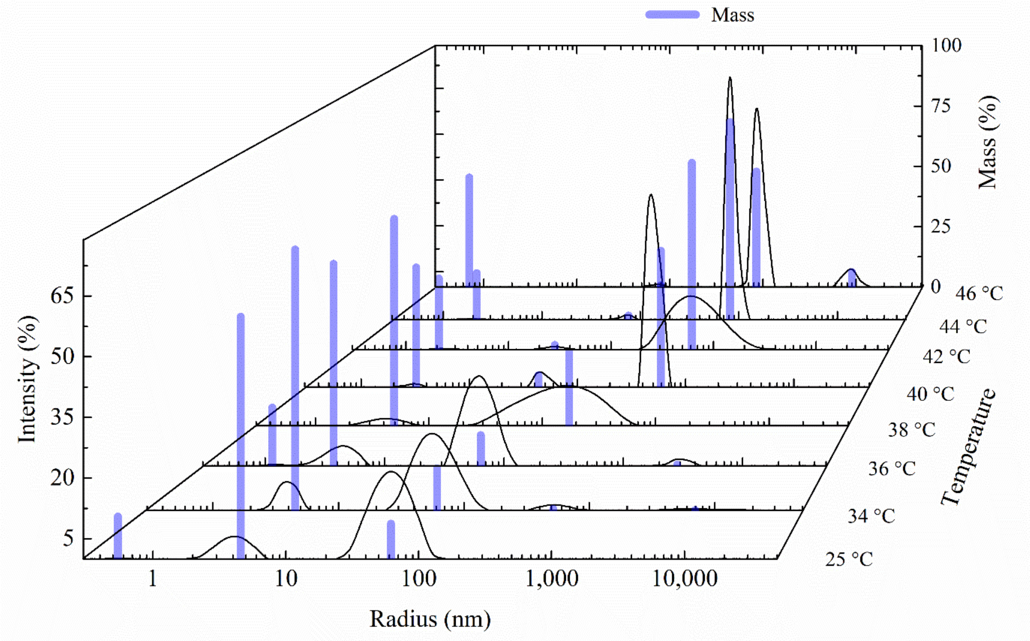

Figure S2. Intensity of light scattered (black traces) and corresponding mass fraction (blue bars) as a function of the hydrodynamic radius at different temperatures. Copolymer B dissolved in water (2 mg mL^{-1}).

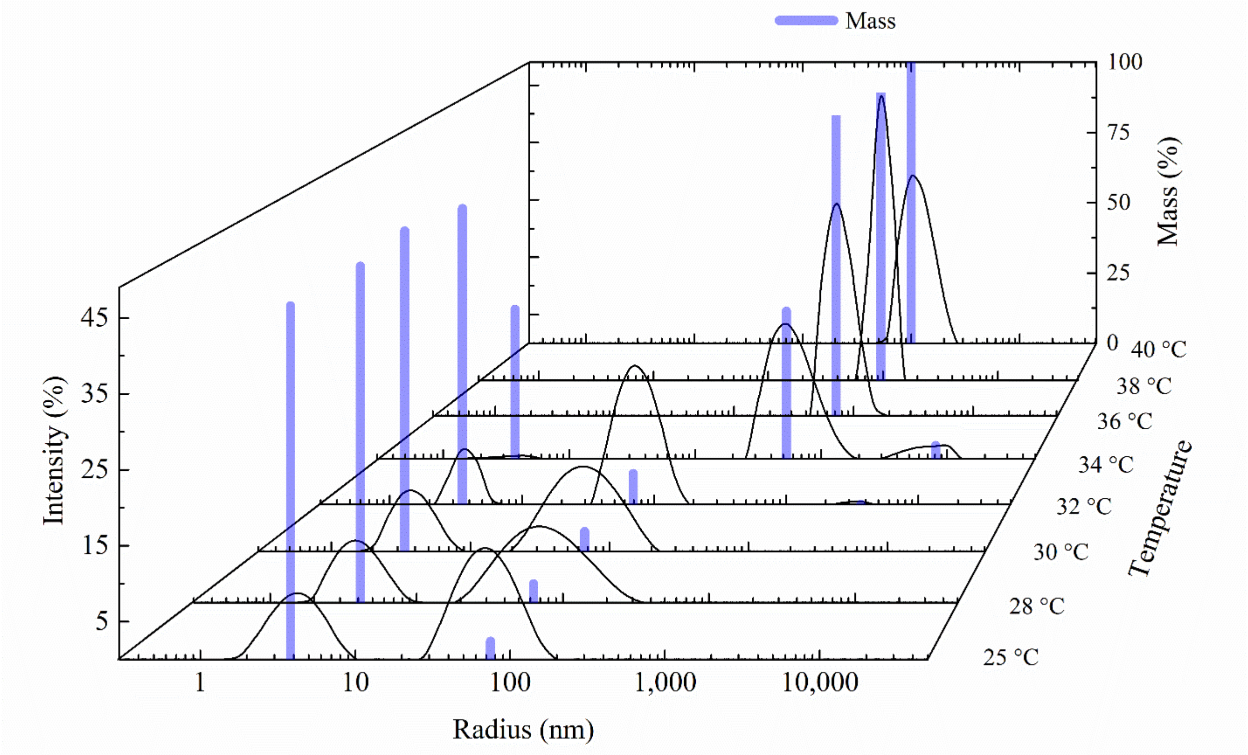

Figure S3. Intensity of light scattered (black traces) and corresponding mass fraction (blue bars) as a function of the hydrodynamic radius at different temperatures. Copolymer C dissolved in water (2 mg mL^{-1}).
